# Complete Genome Sequence of *Spiroplasma* sp. NBRC 100390

**DOI:** 10.1128/genomeA.00008-17

**Published:** 2017-03-09

**Authors:** Mindia Haryono, Wen-Sui Lo, Gail E. Gasparich, Chih-Horng Kuo

**Affiliations:** a Institute of Plant and Microbial Biology, Academia Sinica, Taipei, Taiwan; b Molecular and Biological Agricultural Sciences Program, Taiwan International Graduate Program, National Chung Hsing University and Academia Sinica, Taipei, Taiwan; c Graduate Institute of Biotechnology, National Chung Hsing University, Taichung, Taiwan; d Department of Biological Sciences, Towson University, Towson, Maryland, USA; e College of Arts and Sciences, Salem State University, Salem, Massachusetts, USA; f Biotechnology Centre, National Chung Hsing University, Taichung, Taiwan

## Abstract

*Spiroplasma* sp. NBRC 100390 was initially described as a duplicate of *S. atrichopogonis* GNAT3597^T^ (=ATCC BAA-520^T^) but later found to be different in the 16S rDNA sequences. Here, we report the complete genome sequence of this bacterium to establish its identity and to facilitate future investigation.

## GENOME ANNOUNCEMENT

The bacterial strain *Spiroplasma* sp. NBRC 100390 deposited in the National Institute of Technology and Evaluation–Biological Resource Center (NBRC) was initially described as a duplicate of *S. atrichopogonis* GNAT3597^T^ (1). However, subsequent investigations revealed that the 16S rDNA sequence of this strain (GenBank no. AB681165.1) shows only 96% nucleotide sequence identity to that of *S. atrichopogonis* GNAT3597^T^ maintained in Gail Gasparich’s laboratory at Towson University (GenBank no. KR349130.1) and another duplicate deposited in the American Type Culture Collection (ATCC BAA-520^T^; GenBank no. KR349131.1) (2). To establish the identity of this strain and to facilitate future comparative genomics of *Spiroplasma* species (3, 4), we determined the complete genome sequence of *Spiroplasma* sp. NBRC 100390.

The procedures for sequencing, assembly, and annotation were based on our previous studies of *Spiroplasma* genomes (2, 5–7). We utilized the Illumina MiSeq platform to obtain 301-bp sequencing reads from one paired-end library with approximately 300-fold coverage. Our preliminary analysis revealed that the sequences are almost identical to *Spiroplasma* sp. TU-14 (7). Thus, we chose a resequencing approach using the *Spiroplasma* sp. TU-14 genome (GenBank no. CP017658.1) as the reference. The raw reads were mapped to the reference using the Burrows–Wheeler alignment (BWA) tool version 0.7.12 (8), programmatically checked using the MPILEUP program in SAMtools package version 1.2 (9), and visually inspected using the Integrative Genomics Viewer (IGV) version 2.3.67 (10). The programs RNAmmer (11), tRNAscan-SE (12), and Prodigal (13) were used for gene prediction. Additionally, the presence of putative clustered regularly interspaced short palindromic repeats (CRISPRs) was checked using CRISPRFinder (14).

A total of 18 polymorphic sites were found between *Spiroplasma* sp. NBRC 100390 and the reference, including 11 1-bp deletions, four 1-bp insertions, two single-nucleotide polymorphisms (SNPs), and one 12-bp deletion. All polymorphic sites were manually inspected using Artemis (15) to check if the polymorphisms affected genic regions. We found that all 15 1-bp indels were located in homopolymeric intergenic regions, while the two SNPs both resulted in a nonsynonymous substitution in the predicted functional domain of the respective genes (locus tags S100390_v1c06730 and S100390_v1c09590). However, the BLASTp (16) searches against the NCBI NR database (17) showed that these polymorphisms did not affect the protein domain identification. Finally, the 12-bp deletion was located outside of the predicted functional domain (locus tag S100390_v1c07060). In conclusion, the gene content of *Spiroplasma* sp. NBRC 100390 is identical to that of *Spiroplasma* sp. TU-14 (7), and we annotated the gene names and product descriptions entirely based on the reference genome.

The circular chromosome of *Spiroplasma* sp. NBRC 100390 is 1,199,621 bp in size and has a G+C content of 28.7%; no plasmid was found. The first version of annotation includes one set of 16S-23S-5S rRNA genes, 32 tRNA genes (covering all 20 amino acids), 1,036 protein-coding genes, and four pseudogenes. No putative plectrovirus-related sequence or CRISPR element was found.

### Accession number(s).

The complete genome sequence of *Spiroplasma* sp. NBRC 100390 has been deposited at DDBJ/EMBL/GenBank under accession number CP018022.
